# Editorial: Trends in neuroimmunology: cross-talk between brain-resident and peripheral immune cells in both health and disease

**DOI:** 10.3389/fimmu.2024.1442322

**Published:** 2024-06-18

**Authors:** Shashank K. Maurya, Janina E. Borgonovo, Suryanarayan Biswal, Verónica Martínez-Cerdeño, Rajnikant Mishra, Estela M. Muñoz

**Affiliations:** ^1^ Biochemistry and Molecular Biology Laboratory, Department of Zoology, Faculty of Science, University of Delhi, Delhi, India; ^2^ Integrative Biology Program, Institute of Biomedical Sciences, Faculty of Medicine, University of Chile, Santiago, Chile; ^3^ Department of Human Genetics and Molecular Medicine, Central University of Punjab, Bathinda, India; ^4^ Department of Pathology and Laboratory Medicine, Institute for Pediatric Regenerative Medicine, Shriners Hospitals for Children of Northern California, and MIND Institute at the UC Davis Medical Center, University of California, Davis School of Medicine, Sacramento, CA, United States; ^5^ Biochemistry and Molecular Biology Laboratory, Department of Zoology, Institute of Science, Banaras Hindu University, Varanasi, India; ^6^ Institute of Histology and Embryology of Mendoza (IHEM), National University of Cuyo (UNCuyo), National Scientific and Technical Research Council (CONICET), Mendoza, Argentina

**Keywords:** neuroinflammation, neurodegeneration, microglia, T cells, B cells

The functional anatomy of organisms is maintained by the coordination of different systems, which often rely on interactions between specialized cells and between macromolecules. The immune system works with the circulatory and lymphatic systems to protect most of the organs. However, some organs are considered immune privileged due to the presence of highly selective and regulated barriers, such as the blood-brain barrier (BBB) within the brain (1). The BBB controls periphery-brain molecule exchange and prevents immune effector cells from entering the homeostatic brain. BBB-associated elements, such as endothelial cells, pericytes, astrocytes, and microglia, potentially can function as antigen-presenting cells (APC). Pathological scenarios that induce dysfunction of the BBB and its associated cells may lead to the infiltration of lymphocytes, crossing over from the blood to the brain. Similarly, traumas can also enable B and T lymphocytes to pass bidirectionally between the central nervous system (CNS) and the periphery, via the meningeal lymphatic vessels, which drain into the cervical lymph nodes. Research in animals and humans has revealed that B and T cells are involved in the progression of neurological diseases (NDs). It has been shown that under certain conditions, T cells establish themselves and become resident in the brain, from where they can exert either beneficial or detrimental effects on brain function. Amazing efforts have been made to further comprehend interactions between brain-specific cells and peripheral immune cells, especially their roles and impact on the onset, progression, and eventual resolution of diverse brain pathologies (2–4). The Research Topic discussed herein represents an effort of Frontiers Media S.A. and the authors of this Editorial to develop another special volume related to the healthy and diseased brain (5–8). This Research Topic, which is available for the scientific community and the public, focuses on understanding the complexity of central immune cells and peripheral immune cells, and their cross-talk mechanisms in diverse CNS pathologies. Eleven peer-reviewed manuscripts including four original articles, six reviews, and one systematic review, encompass this special volume. Seventy-five authors from research laboratories located in six countries: Australia, China, Germany, Japan, United Kingdom, and United States took part in this initiative.

Among the interesting contributions, an *in vitro* study on primary murine glia by Li et al. showed differential substrate-dependent and time-dependent phagocytic behavior and phenotypic plasticity among M0-like (unstimulated), M1-like (pro-inflammatory) and M2-like (anti-inflammatory) microglia subtypes. Although the application of M1/M2 terminology in the microglia field has been dismissed, the coexistence of pro-inflammatory and anti-inflammatory microglial states has been documented, including in circumventricular organs (9–12). Li et al. differentiated cultured glial cells into M1-like and M2-like microglia subtypes by treating them either with granulocyte colony stimulating factor and interferon-gamma (GM-CSF/IFNγ), or with macrophage colony-stimulating factor and interleukin-4 (M-CSF/IL-4), respectively. No supplements were added to obtain M0-like microglia. Phagocytosis assays using *E. coli*-rhodamine particles or IgG-FITC beads revealed different preferences and dynamics for the substrates among the microglia subtypes. M1-like microglial cells engulfed more bacteria particles than beads after 3 hours. The opposite behavior was observed with the anti-inflammatory subtype, where M0-like microglia internalized both substrates equally. The authors reported further differences among the three differentiated microglial phenotypes during incubation with both substrates for 16 hours. M2-like microglia showed discontinuous phagocytosis after 8 hours, while M0-like and M1-like microglial cells continuously internalized substrates with different profiles. One interesting observation after a prolonged exposure for 5 days to either *E. coli* particles or IgG-opsonized beads, was that M1-like states and M0/2-M1 transitions were both enhanced, indicating phenotypic plasticity like it occurs in neurodegenerative conditions (13, 14). The study by Li et al. complements the existing knowledge about microglia diversity and plasticity (15, 16), and it opens therapeutic avenues to intervene in microglia-mediated inflammation and neurodegeneration.

Neumaier et al. reviewed current knowledge and therapeutic potential of midkine (MDK), which is a neurotrophic growth factor with dual functions in the healthy and diseased CNS and periphery (17, 18). Due to its multi-functionality, MDK has been involved in the progression or suppression of numerous CNS-related pathologies including autoimmune disorders, such as multiple sclerosis (MS), brain tumors, acute injuries, and other conditions that imply neuroinflammation and neurodegeneration. In the CNS, MDK is spatio-temporally expressed by oligodendrocytes, astrocytes, and neuronal lineages, and maybe also by microglia in response to inflammatory stimuli. In the periphery, hematopoietic and non-hematopoietic cells can produce MDK. This regulator acts through multimolecular receptor complexes, with protein tyrosine phosphatase ζ (PTPζ) as one of the most established components. In addition, multiple signaling pathways are involved depending on the cellular context, thereby facilitating MDK’s multifaceted functions. Interestingly, the authors discussed the role of MDK as a mediator of the neuro-immune cell-to-cell cross-talk in CNS inflammatory scenarios that involve a dysfunctional or leaky BBB. These conditions facilitate the infiltration of MDK-expressing immune cells from the periphery. The recruitment of peripheral immune actors such as macrophages and T cells, and the impact of MDK-signaling events on CNS-resident cells are also addressed within the context of neoplastic diseases (19). The findings included in this review support the importance of MDK as a mediator of tumorigenesis and inflammatory disorders, irrespective of the tissue and cell type, and they emphasize the need for further research to better understand its mechanisms and biomarker potential in neurodegenerative diseases, such as Parkinson’s and Alzheimer’s diseases (PD and AD, respectively).

Neuroinflammation and neurodegeneration are associated with traumatic spinal cord injuries (SCI), which are highly debilitating pathologies. SCI progresses through various phases: acute (up to 3 days post-injury; dpi), subacute (3–14 dpi), and chronic (more than 14 dpi) stages. Yao et al. investigated differential gene expression profiles and pathways in macrophages and microglia across these SCI phases to pinpoint potential therapeutic targets for SCI. The authors applied bioinformatic analysis to the existing scRNA-seq dataset GSE159638 (total 30,958 cells; https://www.ncbi.nlm.nih.gov/geo/query/acc.cgi?acc=GSE159638), which was generated in a mouse model of thoracic contusion SCI (20). Then, they validated the results in a mouse model of cervical SC hemi-contusion injury [wild type and APOE^-/-^ mice; (21)]. They identified apolipoprotein E (APOE) as a central gene of interest in both macrophages and microglia during the subacute and chronic phases of SCI. These cells exhibited high activity, suggesting a crucial role in regulating SCI-associated inflammation. On the other hand, APOE has been linked to pathways related to debris and dead cell clearance (phagocytosis), lipid metabolism, and lysosomal function (22–24). Subsequent experiments demonstrated that APOE knockout (KO) in mice exacerbated neurological deficits, increased neuroinflammation, and worsened white matter loss after SCI at the cervical level. Following SCI, ultrastructural analysis of the KO mice revealed myelin uptake and accumulation of lipid droplets, lysosomes, and needle-like cholesterol crystals in macrophages and microglia. APOE is vital for cholesterol homeostasis within the CNS (25). Together, these results make APOE and its associates promising therapeutic targets for reducing neuroinflammation and for enhancing recovery after SCI.

Zhang et al. contributed to this Research Topic with a comprehensive review of the impact of inflammation and the involvement of infiltrated regulatory T cells (Treg cells) on neuropathic pain (NP) following spinal cord injury (SCI), as well as the potential of cellular therapeutic interventions in SCI-related conditions. The authors discussed the mechanisms behind inflammation and NP after SCI, which include a plethora of cells and mediators. Glial cells, including astrocytes and microglia, and infiltrated immune cells, such as monocytes, macrophages, B cells, and T cells, are involved in these scenarios. These cells, when activated, release inflammatory mediators including chemokines (e.g., CXCL1 and CXCL2) and cytokines (e.g., TNF-α, IL-1β, and IL-6) (26). These molecules affect neurons through multiple signaling pathways, leading to neurotransmitter and ion channel imbalance, increased neuronal excitability, decreased neuronal inhibition, and boosted pain transmission (27). In particular, the authors reviewed the mechanisms by which astrocytes, microglia, and immunosuppressive Treg cells intervene in the pathogenesis of SCI and the subsequent NP. In fact, the inflammatory response following SCI has been tightly linked to a reduction in the number of Treg cells (28). Finally, this review provides a framework for thinking about strategies and challenges (i.e., cell purity, stability, and functionality) in the application of Treg cell therapy in SCI patients who suffer from neuropathic pain.

Chronic inflammation has been associated with different neurological disorders (NDs). Cumulative evidence showed that the recruitment of peripheral immune cells into the CNS is a common characteristic in various NDs (29–32). Among these neuroinflammatory cells, T helper (Th) 17 lymphocytes play an active role in the pathogenesis of CNS-related diseases. The biology of this CD4^+^ Th cell subtype in NDs is addressed in this volume by Shi et al. Th17 cells and their cytokines (e.g., IL-17A, IL-23, IL-21, IL-6, and IFN-γ) contribute to the disruption of the BBB, promote the infiltration of other immune cells into the CNS, excessively activate microglia, and can cause direct cytotoxic damage to neurons (33). The authors described Th17 lymphocytes, including the signaling pathways that induce their differentiation. They also introduced ND-linked environmental factors that may induce the pathogenic potential of Th17 cells, such as peripheral inflammation, enhanced oxidative stress, and changes in the microbiota or diet that affect the gut-brain axis. Shi et al. further discussed the possible immunopathological mechanisms of Th17 cells in AD, PD, MS, amyotrophic lateral sclerosis (ALS), and major depressive disorder (MDD). Finally, therapeutic strategies targeting Th17 lymphocytes, their associated cytokines, and Th17-related molecular mechanisms to treat neurodegenerative diseases, are also addressed.

Grotemeyer et al. elaborated a detailed summary of the interconnected innate and adaptive immune responses in the context of Parkinson’s disease (PD), which is a ND characterized by neuroinflammation and dopaminergic neurodegeneration (34, 35). Interestingly, they propose a mechanism for how neuroinflammation is triggered in PD. They hypothesized that pathological forms of alpha-synuclein (pαSYN), the key protein in PD, might act as a damage-associated molecular pattern (DAMP) to induce and maintain a pro-inflammatory shift of the immune system, via pattern recognition receptor (PRR)-mediated processes (36). Central (e.g., microglia) and peripheral (e.g., T and B cells) immune cells and their mechanisms in the pathophysiology of PD in humans and animal models (e.g., MPTP, 6-OHDA, viral vector, and preformed fibrils), are discussed. Circulating and infiltrated CD4^+^ and CD8^+^ T cells are among the immune effector cells in PD, and their roles are both beneficial and detrimental. The authors also summarized current clinical trials on anti-inflammatory therapy in PD, focusing on the regulation of glucose metabolism, intestinal microbiota, and oxidative stress. Then, they discussed different signaling pathways associated with inflammation and neurodegeneration, such as the pentose phosphate pathway (PPP) (30) and the renin-angiotensin [-aldosterone] system (RA[A]S) (37), to use them as potential therapeutic targets. Finally, the authors suggested that dopaminergic neurodegeneration could be halted by administrating neuroprotective/anti-inflammatory agents early in the course of PD, before severe symptoms have developed.

The involvement of innate and adaptative immune cells in the pathophysiology of multiple sclerosis (MS), and their regulation by physical exercise, are addressed in this Research Topic by Zong et al. The pathogenesis of this neuroinflammatory and autoimmune disease is driven by the dysfunctional activity of immune cells, including those recruited from the periphery into the CNS (38). Aberrant immune responses damage oligodendrocytes and thus, cause severe demyelination, impaired remyelination, axonal degeneration, and altered neurotransmission (39, 40). This results in a spectrum of motor and non-motor symptoms. The disease has no cure and pharmacotherapy is considered the primary treatment. However, drugs have low efficacy, several side effects, and high costs. Alternative MS-modifying interventions, such as physical exercise, have gained attention as a new therapy to alleviate patients’ symptoms (41, 42). In summary, the authors present morphological, cellular, and molecular evidence from animal models (e.g., EAE and toxin and/or virus-induced demyelination models) and human studies of how this type of adjunctive intervention regulates innate and adaptive immune cells, reducing peripheral immune cell infiltration, and eventually leading to a reduction of the autoimmune responses and their concomitant negative effects in the CNS. The authors focused this review specifically on T cells (e.g., CD8^+^ and CD4^+^ cells, including Th17 and Treg cells), B cells, dendritic cells, neutrophils, microglia/macrophages, and astrocytes. Zong et al. also raised a critical view towards the need to conduct more studies in humans, stratifying patients by gender, disease stage, and type, duration, intensity, and cycle of exercise, to better understand the potential of the physical therapy in treating MS.

Beyond the immunological roles, immune cells can participate in other physiological responses that are essential to maintain the homeostasis of the organisms (43, 44). One instance of this is the interplay between enteric C1q-producing macrophages and the enteric nervous system to regulate neuronal and smooth muscle cell functions and thus, gastrointestinal motility and homeostasis (45). It was previously reported that bidirectional signaling between muscularis macrophages and enteric neurons is necessary to ensure gut peristalsis in healthy mice (46). Macrophage-derived bone morphogenetic protein 2 (BMP2) and neuronal colony stimulatory factor 1 (CSF1) are involved in this cross-talk mechanism. In this context, Yip et al. contributed to this Research Topic with an original article in which they studied the participation of CD163 intestinal macrophages and inhibitory interneurons of the myenteric plexus in the regulation of colonic motility. They used a conditional KO *Cx3cr1* (chemokine receptor)*-Dtr* (diphtheria toxin receptor) rat model to transiently deplete resident macrophages in combination with the nitric oxide synthase (NOS) inhibitor NOLA (Nω-nitro-L-arginine), and *ex vivo* video imaging (47, 48). The authors showed that the resident intestinal macrophages are crucial in regulating colonic motility in the absence of the inhibitory neuronal input driven by NO. Whereas, under control conditions, these macrophages might not be relevant. They also showed that these immune cells are important in maintaining healthy intestinal structure. The authors highlighted CD163-positive intestinal macrophages as a potential therapeutic target for gastrointestinal disorders in which inhibitory neuronal input is impaired, such as gastroparesis and achalasia (49, 50). However, Yip et al. pointed out the need for further research to dissect the cell subtypes and to investigate the mechanisms of these functions.

Viengkhou and Hofer reviewed the dual pivotal roles of type I interferons (IFN-Is) in regulating cellular and molecular homeostasis within the CNS, as well as inflammation and immunity associated with diverse NDs, from chronic infections and autoimmune conditions to trauma, aging, and neurodegeneration. Some of these conditions are known as interferonopathies. The authors initially discussed mechanisms by which levels of IFN-Is are altered, especially those mediated by innate immune sensors (e.g., cyclic GMP-AMP synthase/STING signaling pathway), by genetic alterations (e.g., trisomy 21 and mutations in *USP18* or *ISG15*), and by therapeutic interventions for diseases like chronic viral infections, MS, and certain cancers (51, 52). They further presented the canonical and non-canonical IFN-I signaling pathways that imply binding to cell surface receptors and activation of distinct response phases, including an early widespread protein phosphorylation stage and changes in the expression of several IFN-regulated genes (IRGs) (53). The classic path involves the interferon-stimulated gene factor 3 (ISGF3) complex (54), which consists of the transcription factors STAT1 (signal transducer and activator of transcription 1), STAT2, and interferon regulatory factor 9 (IRF9). Then, Viengkhou and Hofer focused the review on the specific responses to IFN-Is mounted by each cell type in the CNS, especially those mediated by neurons, glial cells, and BBB-associated cells. Understanding the diversity in cell responses has been facilitated by single-cell technologies. Moreover, it has been accepted that a diverse spectrum of cellular response states coexists within the diseased CNS, instead of a single prevalent response. Neurons respond to limit the impact of viral infections, but they can suffer neurotoxic effects from increased IFN-I signaling, including fewer dendrites, impaired neurogenesis, and altered neurotransmission (55). Although basal IFN-I signaling in astrocytes is crucial for brain health (56), its contribution to IFN-I neurotoxicity seems yet unclear. A small IFN-I-hyperresponsive microglia subset was identified by single-cell sequencing, which has been associated with age-dependent cognitive decline and synaptic stripping (57, 58). Due to that chronic inflammation has been related to NDs and that IFN-I therapy has been shown to have adverse effects, the authors finished their review discussing the implications and mechanisms of IFN-Is in cerebral interferonopathies, such as Aicardi-Goutières Syndrome (AGS) and chronic viral encephalopathies, as well as in aging, and in diseases with abnormal protein aggregation, including AD and PD (59–61). The authors pointed out that understanding the complexity of IFN-I responses in the CNS is critical for developing targeted therapies for neurological disorders that occur with IFN-I dysregulation. These therapies should consider factors such as cell type, signaling duration, and disease context.

Sun et al. conducted a Mendelian randomization (MR) study to explore the causal relationship between immune cell surface antigens and post-stroke functional outcomes, and to identify novel biomarkers and therapeutic targets for ischemic stroke. The authors employed Genome-Wide Association Studies (GWAS) summary statistics for a two-sample MR analysis, followed by several alternative methods and sensitive approaches. They sourced genetic variants linked to immune cell surface antigens (measured by median fluorescence intensities, MFIs) from the publicly available GWAS catalog (62); outcome data from the Genetics of Ischemic Stroke Functional Outcome (GISCOME) network (63, 64), and statistics about the risk of ischemic stroke from the MEGASTROKE consortium (65). The cohorts were primarily of European ancestry, aged 18 and above. A total of 389 MFIs with surface antigens were included in seven panels (maturation stages of T cell, Treg cell, TBNK, DC, B cell, monocyte, and myeloid cell, respectively). The authors identified genetic variants including single nucleotide polymorphisms (SNPs) associated with MFIs of immune cell surface markers, as measured from samples of peripheral blood. They meticulously selected SNPs that were strongly linked to markers and less likely influenced by non-genetic factors like lifestyle, and they treated them as instrumental variables (IVs) for the MR analysis (66, 67). After a comprehensive analysis, Sun et al. identified 13 suggestive immune cell surface antigens that appear to be associated with post-stroke outcomes. Notably, elevated levels of CD20 on switched memory B cells and of PDL-1 on monocytes appeared to be linked to worse stroke outcomes and severity. In contrast, surface antigen CD25 on CD39^+^ resting Treg cells was found to be associated with favorable post-stroke functional outcomes, possibly due to enhanced Treg cell survival supported by IL2 affinity (68). CD39 was highlighted for its immunosuppressive role, which may be crucial for long-term immune balance after stroke (69). The authors discussed limitations of their analysis including those related to the nature of the sourced data. Overall, this study uncovers potential novel biomarkers and therapeutic strategies targeting immune cell surface antigens to enhance post-stroke recovery, and it warrants further exploration and validation across diverse populations and stroke subtypes.

Considering that research in Treg cells in NDs continues to be a topic of interest (70, 71), Gao et al. contributed to this Research Topic with a bibliometric analysis of the field, spanning from 1991 to 2023, and including 2,739 documents between articles and review articles from the Web of Science Core Collection. The authors used Tableau Public, VOSviewer, and CiteSpace software to perform the study. The research course was categorized into three phases: 1991–2003 (early stage), 2004–2019 (rapid expansion period), and 2020–2023 (fluctuating yet productive phase). A total of 85 countries/regions investigating Treg cells in NDs were identified with the United States, China, and Germany leading in document output. Collaboration among countries/regions was widespread, again with the United States cooperating most (with 57 countries/regions). Notably, Harvard Medical School showed exceptional productivity, citations, link strength, and centrality, reflecting its prolific research and collaborations. Studies examining Treg cells in NDs were published in 859 journals. Among them, the top 11 journals contributed 618 documents, with Frontiers in Immunology, Journal of Immunology, and Journal of Neuroinflammation as the most prominent publishers. The associations of high-frequency keywords, such as “multiple sclerosis”, “inflammation”, and “regulatory T cells”, were found to change throughout the research evolution. Initially, they appeared linked with neuroprotection, neuroimmunology, and immunoregulation (2014), and currently, they shifted towards ischemic stroke, gut microbiota, and the gut-brain axis. Gao et al. identified the top 10 most-cited documents, with three emphasizing the roles of cytokines in autoimmune neurological diseases (72–74), and others examining gut microbiota impact on immune responses and the influence of tumor microenvironment in tumorigenesis (75). Although the United States has led in document output and citations, China emerged as a significant contributor, rising to the forefront in 2022. The study conducted by Gao et al. acknowledges limitations such as language barriers and publication bias, but it emphasizes the need for continual updates to reflect ongoing scientific avenues. This review provides valuable insights for shaping future research directions and therapeutic strategies in this dynamic field.

Overall, the original research and review articles on this Research Topic illustrate the complexity behind the participation of immune cells in the healthy and diseased central nervous system. We expect this Research Topic will encourage researchers to continue their efforts to further investigate immunity and the brain, with the ultimate hope of finding not only new knowledge but also potential clinical interventions to prevent or ameliorate the devastating consequences of neurological diseases.

## Author contributions

SM: Writing – review & editing, Writing – original draft, Conceptualization. JB: Writing – review & editing, Writing – original draft. SB: Writing – review & editing, Writing – original draft. VM: Writing – review & editing. RM: Writing – review & editing. EM: Writing – review & editing, Writing – original draft, Conceptualization.
